# Congenital Thoracic Malformations: A Single-Center Retrospective Study From Hong Kong

**DOI:** 10.7759/cureus.83826

**Published:** 2025-05-10

**Authors:** Chung Yan Michelle Lam, Shu Yan David Lam, Kin-Hoi Thung

**Affiliations:** 1 Department of Paediatrics and Adolescent Medicine, Tuen Mun Hospital, Hong Kong, HKG; 2 Department of Surgery, Tuen Mun Hospital, Hong Kong, HKG

**Keywords:** bronchogenic cyst, bronchopulmonary sequestration, congenital lobar emphysema, congenital pulmonary airway malformations, congenital thoracic malformations, lung malignancies, pleuropulmonary blastoma

## Abstract

Background

Congenital thoracic malformations (CTMs) are a group of developmental lung anomalies, with congenital pulmonary airway malformation (CPAM) being the most prevalent subgroup of all. There had been controversies over the management approaches in asymptomatic CTMs. The study aims to review the clinical course, management options, and outcomes of CTMs in a Chinese population to provide guidance in the future management of the disease.

Methods

This retrospective study reviewed electronic medical records of Tuen Mun Hospital, Hong Kong, from 2002 to 2024 to include all cases with the diagnosis of CTM confirmed through computed tomography (CT). Cases were categorised into the antenatally diagnosed (AN) group and postnatally diagnosed (PN) group for further analysis.

Results

The cohort analyzed 45 cases, of which 18 belonged to the AN group and 27 belonged to the PN group. The majority of patients in the AN group remained asymptomatic throughout follow-up (61.1%); 48.2% of the PN group presented as incidental findings. Pneumonia was the most common complication, affecting 33.3% of the AN group and up to 59.3% of the PN group. Most of the patients had a single uncomplicated pneumonia (AN group: 83.3%; PN group: 75%). Surgeries were performed in 44.4% of the AN group and 66.7% of the PN group, with notable postoperative complications observed (AN: 25%, PN: 22.2%). Importantly, only one case of lymphoepithelioma-like carcinoma in a patient with bronchopulmonary sequestration (BPS) was identified, and no malignancies were found in other CTM entities.

Conclusion

In view of the relatively indolent and benign course found in this study, a conservative approach with surgery at a later age could be an alternative to early surgery in asymptomatic CTMs.

## Introduction

Congenital thoracic malformation (CTM) is a term to describe a group of developmental lung lesions, including congenital pulmonary airway malformation (CPAM) [1-6], bronchopulmonary sequestration (BPS), bronchogenic cyst, congenital lobar emphysema (CLE), and bronchial atresia. It is a rare disease entity affecting up to 30-42 in 100,000 individuals each year. Although most of them can be clinically asymptomatic throughout life, some lesions can cause severe respiratory symptoms and complications at different ages. CPAM is the commonest type amongst them and can be further assigned into one of the five subtypes under the Stocker Classification. Different CTMs were also known to be associated with different lung tumours, including pleuropulmonary blastoma (PPB) [7-10], adenocarcinoma, and squamous cell carcinoma.

Due to technological advancements over the years, more CTMs were diagnosed antenatally, and the majority remained asymptomatic at birth. Although many had advocated early surgical resections of these lesions, there had been ongoing debate over a standardised management approach in initial asymptomatic CTMs owing to the disease rarity, wide range of presentations, and resources available across different centers.

There were reviews from Caucasian literatures to discuss the clinical presentations and outcomes of the disease [2,3,11-14], yet there were fewer reviews in Asia or locally [1,8,15-17], and even scantier reviews on those being diagnosed after birth. Our review included Chinese patients to fill in the knowledge gaps and guide the establishment of updated management algorithms for the disease in Chinese populations.

The study aims to delineate the incidence, clinical characteristics, management options, and outcomes of patients with CTMs diagnosed antenatally and after birth.

## Materials and methods

Study design

This retrospective observational cross-sectional study was conducted at the Department of Paediatrics and Adolescent Medicine of Tuen Mun Hospital (TMH), Hong Kong; a tertiary public hospital serving a population of over 1.1 million (15% of the total Hong Kong population) [18].

Inclusion and Exclusion Criteria

Cases were identified through the Clinical Data Analysis and Reporting System, the system incorporates all electronic medical records of the hospital. All patients diagnosed between 2002 and 2024 with International Classification of Diseases, Ninth Revision (ICD-9) codes 748.4 ("Congenital cystic lung"), 748.69 ("Other congenital anomalies of lung"), 748.52 ("Sequestration of lung"), 748.56 ("Extralobar bronchopulmonary sequestration"), 748.57 ("Intralobar bronchopulmonary sequestration"), and 770.23 ("Lobar emphysema of newborn") were included in the study. All CTMs diagnoses were confirmed by computed tomography (CT).

Patients suspected to have CTMs on initial antenatal ultrasound (USG) or postnatal chest X-rays (CXR), yet subsequently exhibited no lung lesions upon CT evaluation, were excluded.

Clinical data and outcome measures

Medical records of the included subjects were reviewed to analyse their baseline clinical characteristics, including the age of diagnosis, gender, CTM subtypes, and lobe involvements.

Primary outcomes were the mortality rate, incidence of PPB and incidence of other lung malignancies; while secondary outcomes focused on various pulmonary morbidities including chronic cough, pneumonia, hemoptysis, pleural effusion and pneumothorax; as well as surgical operation rate, related complications, the number of CT performed before surgery; and pathological findings of operated cases.

Cases were further categorised into the antenatally diagnosed group (AN group) and postnatally diagnosed group (PN group) for analysis. The AN group consists of patients diagnosed to have CTMs through antenatal USG and confirmed by CT after birth. The PN group includes cases diagnosed after birth and not known antenatally.

Statistical analysis

In this study, continuous variables, including the age at diagnosis, age at surgery, duration of follow-up, etc., were analyzed and reported either as means with standard deviations (mean ± SD) for normally distributed data or as medians with interquartile ranges (IQR) for non-normally distributed data. The IQR represents the difference between the 25th percentile (first quartile) and the 75th percentile (third quartile) of the data distribution.

CT findings were systematically reviewed to determine the prevalence of various subtypes of CTMs. The incidence of pneumonia associated with different CTM subtypes was thoroughly analysed to identify any significant patterns or associations.

The operation rate and rate of operative complications were also evaluated. All discrete variables (e.g., incidence, prevalence, operation rates, and complication occurrences) were expressed as absolute counts (n) or percentages (%).

Ethical approval

The study was approved by the Central Institutional Review Board (IRB) of the Hospital Authority, Hong Kong. The IRB approval number is PAED-2024-049.

## Results

Diagnosis and initial clinical presentation

From 2002 to 2024, 45 cases were diagnosed with CTM. Forty percent were diagnosed antenatally (n=18) while 60% were diagnosed after birth (n=27). The male-to-female ratio is 1.05:1.

Patients’ Characteristics

Among those diagnosed antenatally, the mean diagnostic age was 22.4±5.6 weeks of gestation, which correlated with the time of morphology scan. Nine of them were males and nine of them were females. None of them had a fetal MRI performed.

The median follow-up time is 6.3 years after birth (IQR 6.2). One of them (5.6%) was lost to follow-up after one consultation at the outpatient clinic (Table 1).

**Table 1:** Patient characteristics in antenatal (AN) and postnatal (PN) groups of congenital thoracic malformations (CTMs) CPAM: Congenital pulmonary airway malformation; BPS: Bronchopulmonary sequestration; PS: Pulmonary sequestration; EL: Extralobar; IL: Intralobar; CLE: Congenital lobar emphysema; IQR: Interquartile range

Characteristic	AN group	PN group
Male: Female ratio	1:1	1.08:1
AN diagnostic age (gestational week)	22.4±5.6	-
PN diagnostic age (years old)	-	29±24.4
Median follow-up time (years)	6.3 (IQR=6.2)	4.4 (IQR=7.3)
CTM subtypes	(n=18)	(n=27)
CPAM - Stocker type I	7 (38.8%)	11 (40.7%)
CPAM - Stocker type II	6 (33.3%)	1 (3.7%)
CPAM + PS - Hybrid	0 (0.0%)	1 (3.7%)
BPS - EL	3 (16.7%)	2 (7.4%)
BPS - IL	1 (5.6%)	7 (26.0%)
Bronchogenic cyst	1 (5.6%)	2 (7.4%)
CLE	0 (0.0%)	3 (11.1%)

For the 27 patients diagnosed after birth, 14 were male and 13 were female. The mean age of diagnosis was 29±24.4 years. Eleven (40.7%) of them were diagnosed in childhood or the adolescent period. The median follow-up time was 4.4 (IQR 7.3) years. A total of 14.8% (n=4) was lost to follow-up.

CPAM was the commonest subtype of CTM in both groups (overall prevalence 55.6%), predominantly type I lesion (40% of all CTMs), followed by BPS as the second most prevalent subtype (28.9%). The prevalence of different subtypes in the AN and PN groups was listed in Table 1.

All CTM cases were unilateral lesions. The left lower lobe was the most commonly involved lobe if all entities were taken together. As shown in Table 2, the right upper lobe (28% of all CPAM) and right lower lobe (24% of all CPAM) were the two most commonly involved lobes in cases with CPAM. Three CTM cases involved more than one lobe. The hybrid case was in the left lower lobe. Almost all cases of BPS involved the left lower lobe (92.3% of all BPS). For bronchogenic cyst, there was one case each at the right middle lobe, left lingula, and subcarinal region, respectively. Two CLE cases were in the left upper lobe, and one was in the right middle lobe.

**Table 2:** Lobe involvement in different CTM subtypes CTM: Congenital thoracic malformation; CPAM: Congenital pulmonary airway malformation; BPS: Bronchopulmonary sequestration; CLE: Congenital lobar emphysema; RUL: Right upper lobe; RML: Right middle lobe; RLL: Right lower lobe; LUL: Left upper lobe; LL: Left lingula; LLL: Left lower lobe

Lobe involvement in different CTM subtypes	n (%)
CPAM (type I and II) (n=25)	
RUL	7 (28.0)
RML	0 (0.0)
RLL	6 (24.0)
LUL	5 (20.0)
LL	1 (4.0)
LLL	3 (12.0)
RML + RLL	1 (4.0)
RUL + RML	1 (4.0)
LUL + LL	1 (4.0)
Hybrid lesion - LLL (n=1)	100.0%
BPS (n=13)	
RUL	1 (7.7)
LLL	12 (92.3)
Bronchogenic cyst (n=3)	
RML	1 (33.3)
LL	1 (33.3)
Subcarinal	1 (33.3)
CLE (n=3)	
RML	1 (33.3)
LUL	2 (66.7)

Primary Outcomes

No cases died or had malignancies during the follow-up period in the AN group. In the PN group, two (7.4%) of them died due to other diseases. One (3.7%) case had lymphoepithelioma-like carcinoma with extralobar pulmonary sequestration. The patient presented with a chronic cough and was diagnosed at 59 years old. She died of the disease three years after the operation due to recurrence at the surgical bed with multiple metastases.

*Secondary Outcomes:* *Pulmonary Morbidities*

In the AN group, more than half of them remained asymptomatic during the follow-up period (61.1%, n=11), six developed pneumonia (33.3%), and one presented as respiratory failure after birth due to the space-occupying effect of the CTM (5.6%).

As for the PN group, they mostly presented as an incidental finding during body check (48.2%, n=13) or pneumonia (37.0%, n=10). There were three cases of hemoptysis (11.1%) and one case of pneumothorax (3.7%) as initial presentation. Details are shown in Table 3.

**Table 3:** Pulmonary morbidities in patients with CTMs CTM: Congenital thoracic malformation; AN: antenatal; PN: postnatal

Pulmonary morbidity	AN group (n=18)	PN group (n=27)
Asymptomatic/incidental findings	11 (61.1%)	13 (48.2%)
Pneumonia	6 (33.3%)	10 (37.0%)
Pneumothorax	0 (0.0%	1 (3.7%)
Haemoptysis	0 (0.0%)	3 (11.1%)
Respiratory failure - space-occupying effect	1 (5.6%)	0 (0.0%)

Pneumonia was the most common pulmonary morbidity in both groups. In the AN group with pneumonia, five (83.3%) of them suffered from one episode of pneumonia, while the remaining one (16.7%) case had five episodes of pneumonia. The mean age of their first presentation was 4.5±3.8 years. The case with recurrent pneumonia had an episode of pneumonia complicated by a mycobacterium abscess within the malformed lesion.

In the PN group, a total of 16 (59.3%) patients had pneumonia either as initial presentation or during their follow-up period. The mean age of their first presentation of pneumonia was 18.2±19.5 years. Most of them (n=12, 75%) had a single episode of pneumonia, while four (25%) had recurrent pneumonia. Three out of 16 had complicated pneumonia requiring chest drain insertion due to pleural effusion (n=2, 66.7%) and large pneumothorax (n=1, 33.3%). One (6.3%) case had severe pneumonia requiring humidified high-flow oxygen support.

More than half (60%) of all CPAM developed pneumonia, with up to 83.3% in the CPAM of the PN group. Only two cases of all BPS (15.3%) had pneumonia. All three cases of CLE and the single case of hybrid lesion had pneumonia (100%), and the incidence of pneumonia in bronchogenic cysts in all CTM was 33.3%. Table 4 shows the overall and individual incidence of pneumonia in different CTM subtypes in the two subgroups.

**Table 4:** Incidence of pneumonia in different CTM subtypes in AN and PN groups CTM: Congenital thoracic malformation; CPAM: Congenital pulmonary airway malformation; PS: Pulmonary sequestration; BPS: Bronchopulmonary sequestration; CLE: Congenital lobar emphysema; AN: antenatal; PN: postnatal

CTM subtypes	AN group	PN group	Overall incidence
CPAM (AN group: n=13; PN group: n=12)	5 (38.5%)	10 (83.3%)	15 (60.0%)
CPAM + PS - Hybrid lesion (AN group: n=0; PN group: n=1)	-	1 (100%)	1 (100.0%)
BPS (AN group: n=4, PN group: n=9)	1 (25.0%)	1 (11.1%)	2 (15.4%)
Bronchogenic cyst (AN group: n=1, PN group: n=2)	0 (0.0%)	1 (50%)	1 (33.3%)
CLE (AN group: n=0; PN group: n=3)	-	3 (100.0%)	3 (100.0%)

In both groups of patients, pneumonia mostly occurred in the same lobe with its congenital lesions (AN group: n=5, 83.3%; PN group: n=14, 87.5%), reflecting that pneumonia was highly associated with CTMs.

Operation

A total of 27 patients had operations performed. Details of the patients who had been operated on are shown in Table 5.

**Table 5:** Characteristics in patients with CTM and operation performed CTM: Congenital thoracic malformation; AN: antenatal; PN: postnatal

Parameter	AN group	PN group
Cases with an operation performed	8 (44.4%)	18 (66.7%)
Mean age of operation (years)	7.3±7.9	34.2±21.8
Mean n umber of CT scans prior to operation	1.87±2.1	2.16±1.2
Cases with postoperative complications	2 (25%)	4 (22.2%)

In the AN group, eight (44.4%) were operated on. The mean age of operation was 7.3±7.9 years, with the youngest being operated on at seven months old. The average number of CTs performed before operation was 1.87±2.1. Two (25.0%) had surgeries done as primary prevention, while six (75.0%) of the others had operations performed as secondary prevention of re-infection.

In the PN group, 18 (66.7%) cases were operated on. The mean age of operation was 34.2±21.8 years. On average, they had an average of 2.17±1.2 CTs performed prior to the surgery. The reasons for surgery were (a) primary prevention before any complications (n=5, 27.8%); (b) secondary prevention for re-infection or recurrence of other complications (e.g. pneumothorax, hemoptysis) (n=11, 61.1%); (c) emergency operation as treatment in acute episodes of complicated disease (pneumothorax and lung abscesses) (n=2, 11.1%).

The postoperative complication rates were similar in both groups (AN group: n=2, 25%; PN group: n=4, 22.2%), and there were a few cases with significant complications in both groups. One case in the AN group (12.5%) had pneumothorax recurrence after initial complete expansion of the operated lung, one week after discharge, requiring chest drain re-insertion. The drain was removed after 10 days with no further pneumothorax.

In the PN group, one (5.6%) had a significant persistent air leak requiring chest drain re-insertion, while two had significant bleeding (n=2, 11.1%). One of the bleedings occurred intraoperatively, resulting in severe hemodynamic compromise in need of cardiac massage; the patient subsequently suffered from long-term neurological disability due to the hypoxic injury. The other patient had postoperative bleeding requiring re-thoracotomy to stop the bleeding.

Pathological diagnosis

The final histopathological diagnosis was well correlated with the initial CT diagnosis in both groups. This reflects the important role of imaging in diagnosing CTMs.

## Discussion

This study provides important insights into CTMs in a Chinese population. By analysing both antenatally and postnatally diagnosed cases, it highlights the clinical course, complications, and management strategies, emphasizing the largely benign nature of the disease and the need for individualized management.

The median follow-up time of both groups was beyond three years, allowing the analyses of the development of various complications to be more representative.

Key findings

In the AN group, the majority of patients remained asymptomatic throughout follow-up (61.1%), whereas nearly half of the patients in the PN group presented as an incidental finding (48.2%) at diagnosis. The majority of the PN group were diagnosed after reaching adulthood (59.3%). All these findings underscore the indolent nature of CTMs, with many lesions remaining stable and clinically silent for long periods [3,10].

Pneumonia was the predominant pulmonary morbidity in both groups, which mostly occurred as a single uncomplicated episode beyond infancy. Other morbidities, such as pneumothorax and hemoptysis, were rare, which again suggests the disease runs a relatively benign course. Nevertheless, there could be a delayed onset of symptoms in many cases, as demonstrated through a higher proportion of overall pneumonia cases (PN group: 59.3% vs. AN group: 33.3%) and a higher rate of recurrent pneumonia (25.0% vs. 16.7%) in the PN group. The mean age of first pneumonia in the PN diagnosed group was beyond adulthood (18.2±19.5 years), which also illustrated that complications such as infection could manifest over time and highlighted the need for long-term follow-up.

Our cohort had a relatively high incidence rate of pneumonia in the group of CPAM as compared to some previous literature [19]. Nearly all CPAMs in the PN group were subsequently infected (83.3%), implying that those AN CPAMs may have a high chance of developing pneumonia when they grow older. This poses important implications while counselling patients about the infection risks in different CTMs. At the same time, as opposed to previous evidence [20,21], the incidence of pneumonia was relatively low in cases with BPS (15.3%).

The overall malignancy rate was very low, with only one case of lymphoepithelioma-like carcinoma identified in a patient with BPS at the age of 59. No cases of malignancy were observed in cases with CPAM. From personal communication, there are no other lung malignancies other than PBB in the paediatric age group associated with CTMs in Hong Kong as well. These findings are consistent with existing evidence that CTMs mostly run a benign course throughout childhood and adolescence [3,10].

Management approaches

Early Surgery Versus Operation at a Later Age

Our findings support that surgery should be the mainstay of management for symptomatic patients, as up to one-fourth of the cases in the PN group had recurrent pneumonia, and patients in the AN group with recurrent pneumonia required multiple hospital stays due to the persistence of their condition. Patients, therefore, should undergo elective surgery after the first pneumonia to prevent its recurrence.

As for asymptomatic patients, management strategies should be individualised after counselling on the pros and cons of various approaches. Although they could be reassured of the largely benign nature of the disease, they should be aware of the high chance of the emergence of complications such as pneumonia in later life, particularly in cases with CPAM. Besides, the very small yet non-negligible risk of malignancies in different CTMs should also be emphasized [8,22].

The study demonstrated that complications such as pneumonia are more likely to occur later in life, and there was no case of malignancy in childhood. A more conservative approach with follow-up and elective surgery at a later age could be an alternative to early surgery during infancy.

Role of Imaging

The role of imaging for diagnosis and as part of the surveillance during follow-up should then be discussed. CT findings in this cohort correlated strongly with final pathological diagnoses, signifying its effectiveness in diagnosing the lesions and monitoring disease progression. However, this study also highlights the potential significant radiation exposure in patients undergoing repeated CT imaging, particularly in those managed conservatively or awaiting surgery. For instance, some operated patients had four or more CT scans preoperatively, raising concerns about cumulative radiation risk, especially in younger patients. With specific CTM scanning protocols, MRI provides a radiation-free alternative for surveillance of known lesions, especially in asymptomatic or conservatively managed patients [23,24]. It should be considered a primary imaging modality for long-term follow-up when feasible.

Genetic Testing

The concern for malignancy, particularly PPB, has been a driving force behind early surgical intervention in some protocols. A local study had demonstrated a strong association of DICER1 gene mutations with PPB [9]. There is also ongoing research across the world on various genes, such as the KRAS mutation, that may be associated with the malignant transformation of CTMs [25,26]. This raises the possibility of incorporating genetic testing into clinical decision-making to better stratify malignancy risk and guide management in asymptomatic cases in the future.

Limitations

This single-center study is limited by its retrospective nature and relatively small sample size, which constrain the generalizability of its findings. Multi-center studies with larger cohorts would help validate these results and provide more robust evidence. There was also a lack of data on lung function tests to look at the impact of CTM on lung growth.

Future directions

Prospective studies are needed to evaluate the long-term outcomes of both early and late surgical intervention. MRI may be considered as an imaging alternative to follow up complications in the later operative approach, aiming to reduce radiation exposure. The use of genetics studies to identify high-risk cases could further refine the management of CTM. Lung function tests could also play a role in monitoring asymptomatic patients and post-operative patients to provide information on the impact of various management approaches in CTMs.

## Conclusions

This study reinforces that CTM typically follows a benign course, with the majority of patients being asymptomatic or experiencing only mild complications, such as pneumonia. However, the malignant changes in the death case highlight the rare but significant complication of CTM. The removal of CPAM, BPS, and bronchogenic cyst for the prevention of malignant changes cannot be under-emphasized. The risk of surgical complications should also be addressed in both symptomatic and asymptomatic patients. The timing of surgery remains a controversial area, and surgical treatment at a later age could be an option. If a “watch-and-wait” approach is adopted for asymptomatic cases, the risks of emerging complications, including malignancies, should be discussed. Larger prospective studies should be performed in the future to enhance the understanding and management of the disease.
